# Supplementary material for: The Construction and Exploration of a Comprehensive MicroRNA Centered Regulatory Network in Foxtail Millet (Setaria italica L.)
Source: Front Plant Sci. 2022 May 6;13:848474. doi: 10.3389/fpls.2022.848474 (PMC9121102; doi:10.3389/fpls.2022.848474)
Supplement: Supplementary file 4 [file Data_Sheet_4.zip › Sit-miR3979_Seita.9G445200.1_2200_TPlot.pdf]

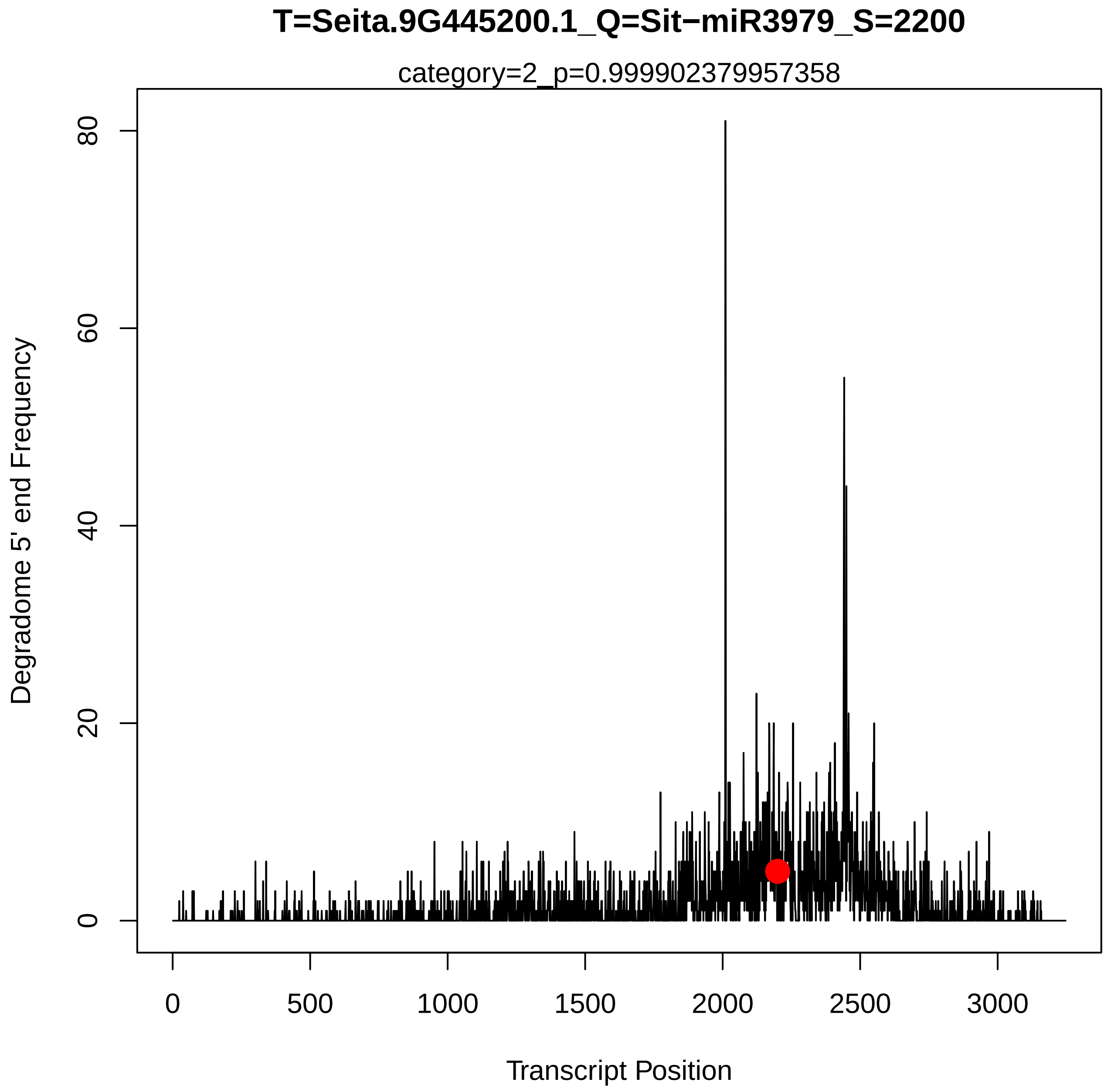

T=Seita.9G445200.1_Q=Sit−miR3979_S=2200
category=2_p=0.999902379957358
Degradome 5' end Frequency
0
20
40
60
80
0
500
1000
1500
2000
2500
3000
Transcript Position
